# PROTOCOL: Day reporting centres for reducing recidivism: A systematic review

**DOI:** 10.1002/cl2.1023

**Published:** 2019-07-19

**Authors:** Joel Miller, Nicole M. Sachs, Marina K. Saad

**Affiliations:** ^1^ School of Criminal Justice Rutgers University Newark New Jersey; ^2^ School of Criminal Justice Fairleigh Dickinson University Teaneck New Jersey

## 1 BACKGROUND

### 1.1 The problem, condition, or issue

The day reporting centre (DRC) model emerged in Great Britain in the 1960s, and was first seen in the U.S. a couple of decades later (Boyle, Ragusa, Lanterman, & Marcus, 2011; Craddock, 2000). Its arrival coincided with a wave of interest in alternative to incarceration programmes that might help limit the costs of jail and prison and reduce overcrowding, while maintaining public safety. DRCs are nonresidential facilities that provide offenders with supervision and forms of rehabilitative programming (Boyle et al., 2011). A primary goal of DRCs is to reduce recidivism. However, thus far, no systematic review has sought to establish the efficacy of these programs in doing this.

### 1.2 The intervention

Offenders participating in DRC programs typically reside at home and report to the DRC on a regular schedule, which can be a couple of times per week or several times per day (Diggs & Pieper, 1994). They provide a higher level of supervision compared with probation and parole (more frequent contacts between offenders and supervisors) while being less restrictive than traditional imprisonment. Rehabilitative components may include cognitive‐behavioural therapies, substance abuse treatment, life skills training, education classes, or community referrals.

Notwithstanding commonalities, DRC programs show some variability. They differ according to the types of offenders and clients serviced, the duration of offenders’ involvement in the programme (see Craddock, 2000; Jones & Lacey, 1999), the frequency of reporting required (Craddock, 2000), and the treatment and services offered. This variety is evident across studies of different DRCs. Thus Boyle et al. (2011) describe a set of DRC programs in New Jersey targeting parolees at risk of revocation because of technical violations of supervision. The program involves an initial 90‐day term, which may be extended, and offers a variety of services, with an emphasis on vocational training and readiness. Meanwhile, Champion, Harvey, and Schanz (2011) describe a DRC program targeting habitual nonviolent offenders on probation supervision with services to address substance abuse problems and a lack of basic living skills. And Jones and Lacey (1999) examined DRC programs in Maricopa County, AZ, targeting adult felony DWI offenders.

### 1.3 How the intervention might work

Existing studies are often agnostic about the ways in which DRC programs might work to reduce recidivism (e.g., Boyle, Ragusa‐Salerno, Lanterman, & Marcus, 2013). However, we suggest four possible mechanisms:

1) Short‐term suppression of offending through deterrence:
  The frequent contact between offenders and supervision staff at DRCs may produce a deterrent effect, as offenders are more often called to account for their daily activities.
2) Short‐term suppression of offending through changes to routine activities: The frequent contact between offenders and supervision staff at DRCs may have important impacts on the offender's routine activities, such that they spend less time in criminogenic environments, where temptations to commit crime are present. This would lead to reductions in offending for the duration of their involvement in the DRC.
3) Longer‐term effects on offending through stabilisation:
  The structure provided by DRCs, over a period of time, may provide the offender a more consistent routine, allowing them to re‐focus their priorities and address challenges, with longer‐term effects on criminal lifestyles.
4) Longer‐term effects on offending through services and treatment: DRCs tend to incorporate service components, such as substance abuse treatment, cognitive behaviour programs, or employment and training support. These services may have direct effects on criminogenic needs, reducing offenders’ propensity to reoffend.

We should also acknowledge two additional effects, the first a possible iatrogenic effect, caused potentially by offenders grouping together at DRCs and providing delinquent reinforcement to one another. Such dynamics may account for short‐term iatrogenic effects on recidivism found by Boyle et al.’s (2013) experimental study of DRCs (see also Dishion, McCord, & Poulin, 1999). The second potential effect is one in which increased contact between supervision staff and offenders at DRCs may lead to increased opportunities to detect violations and recidivism.

### 1.4 Why it is important to do the review

A variety of studies (including those mentioned previously) have sought to assess the effects of DRCs on offending outcomes. While there are few randomised control trials of DRCs, there is at least one: The study by Boyle et al. (2011) of DRCs catering for New Jersey parolees. There are also are a number of quasi‐experimental studies that appear to use credible comparison groups along with statistical controls to assure an adequate level of internal validity (Champion, Harvey, & Schanz, 2011; Jones & Lacey, 1999; Ostermann, 2009; Solomon, 2008). For example, Solomon (2008) used a preprogram cohort to compare with defendants sentenced to a “day custody program” in Manhattan, New York, while Craddock (2000) compares probationers in Wisconsin attending a DRC with a similar group who would have been eligible, but did not participate.

However, no scholars have yet attempted to synthesise the disparate findings of these (and other) studies to form generalised conclusions about DRCs. Specifically, there is to date no known systematic review or meta‐analysis of DRCs. Our proposed systematic review will help fill this gap by assessing whether DRCs appear, overall, to be effective at reducing recidivism, and—if possible—whether effects are contingent on variations in program design and population. In doing so, it will focus on a range of offender types, including both adults and juveniles, and program elements, contingent upon the local context of the DRC program (Diggs & Pieper, 1994; Parent, 1990; also see Jones & Lacey, 1999; G. Martin, 2003; Solomon, 2008). Generally speaking, most DRC programs do not service violent or sex offenders. However, participants range through pretrial releases, violators of probation or parole, probationers, work releases, furloughs, those who have finished their prison sentence and are on mandatory supervision, early‐releases close to parole eligibility, and those released due to overcrowding emergencies (McDevitt & Miliano, 1992; Parent, 1990).

## 2 OBJECTIVES

We will perform a comprehensive review and synthesis of rigorous research available on (a) the short‐term suppression effects of DRC programs on recidivism while participants are attending the DRC, and (b) the longer‐term effects on recidivism during and beyond the period of participation, indicative of broader rehabilitative impacts. The focus of the review is on programs that require offenders to make multiple appearances at a centre each week, and which may also provide rehabilitative programming. The effects of these programs are tested against a counterfactual of offenders not required to attend a DRC, in (a) comparison group(s) of offenders provided that are plausibly similar to DRC participants, or (b) where there are statistical controls that address likely selection biases. We will make separate and combined comparisons between DRC offenders and, respectively, comparison groups under community supervision. The primary outcome measure is recidivism, considered to be rearrest, reconviction, or reincarceration. We are interested in both short‐term and long‐term effects on recidivism.

The research questions we plan to address are:

1. To what extent do day reporting centres influence offenders’ short‐term offending behaviour?
2. To what extent do day reporting centres influence offenders’ long‐term offending behaviour?
3. Which program components or offender characteristics moderate the overall effect of day reporting centres on recidivism?

## 3 METHODOLOGY

### 3.1 Criteria for including and excluding studies

#### 3.1.1 Types of interventions

Eligible studies will test the effects of participation in a DRC on recidivism. An offender is considered to be participating in a DRC if he or she is required to make appearances at physical premises on multiple (more than one) occasions each week; he or she may also receive rehabilitative programming during these visits.

#### 3.1.2 Types of study designs

In an attempt to balance internal validity with coverage, we will include randomized controlled trials, rigorous quasi‐experimental designs involving matching and premeasures and postmeasures of offending behaviour, quasi‐experiments with plausibly similar comparison groups, and quasi‐experiments with credible post hoc statistical controls to compensate for differences between groups. We expect, based on our current knowledge, to find several studies meeting these criteria for inclusion in the systematic review.

The comparison group(s) must be comprised of offenders who are under criminal justice supervision in the community, for example, experiencing conventional probation or parole supervision. Moreover, we will code characteristics of these comparison criminal justice sanctions, such as whether probation or parole supervision is intensive, or whether it incorporates a strong therapeutic component. This will allow us to take into account the nature of the comparison groups in our analyses.

#### 3.1.3 Types of participants

DRCs can serve both juvenile and adult offenders and clients, though we expect most DRCs to serve adult males. While most of these will likely be convicted or adjudicated, pretrial program clients will not be.

#### 3.1.4 Types of outcome measures

Eligible studies will measure recidivism in terms of new arrests, convictions, and/or incarcerations. Technical violations of probation or parole, for example failing a drug test, will be included as a separate outcome measure, though these are not of primary interest. This is because technical violations may vary simply by as a function of being supervised DRCs—where high levels of surveillance could increase the detection of a violation or new offence—even without differences in underlying offender behaviour.

#### 3.1.5 Duration of follow‐up

We will examine outcomes for a minimum of 1 month for short term outcomes. For longer‐term outcomes, we will examine outcomes from at least 6 months. There will be no upper limit to the follow‐up duration.

#### 3.1.6 Types of settings (and timeframes)

Given that DRC programs are relatively new (they emerged in the 1960s; mid‐1980s in the United States), we will consider all studies from 1960 onward for inclusion in the systematic review. Studies will not be excluded on the basis of geography. However, we will limit studies to those written in English.

#### 3.1.7 Search strategy

We will use a number of strategies to conduct an exhaustive search for literature on DRCs. The main literature search will involve keyword searches of online databases and search engines, research organisations, and government databases. We will also review reference lists in reviews of DRCs, and in all studies being considered for inclusion in the review. We will make every effort to locate unpublished material, for example through reaching out to experts in the field. A study's eligibility will be determined by first reading its title and then (if it appears relevant) the abstract, and then reviewing the full text, the latter occurring only if the study appears appropriate.

List of online databases:

1. Google (www.google.com), Google Scholar (https://scholar.google.com/), Google Books (https://books.google.com/)
2. Criminal Justice Abstracts (via EBSCOhost)
3. Academic Search Premier (via EBSCOhost)
4. Proquest Dissertations and Theses (ProQuest)
5. Rutgers Grey Literature Database (https://njlaw.rutgers.edu/cj/grey/)
6. JSTOR
7. PsycINFO (via Ovid)
8. Australian Criminology Database (CINCH)
9. Government Publications Office Monthly Catalogue
10. International Bibliography of the Social Sciences (via ProQuest)
11. National Criminal Justice Reference Service (NCJRS) Abstracts
12. 12 Sage Full Text Collection: Criminology and Criminal Justice
13. Sage Full Text Collection: Sociology
14. Sociological Abstracts (via ProQuest)
15. Social Sciences Premium Collection (via ProQuest)
16. Social Sciences Citation Index (via Web of Science)
17. All Academic

List of Research Organisations and Government Department Websites:

1. American Correctional Association
2. American Probation and Parole Association
3. Home Office Research, Development, and Statistics Archive (UK)
4. International Community Corrections Association
5. Ministry of Justice (UK)
6. National Association for the Care and Resettlement of Offenders (UK)
7. National Institute of Corrections (USA)
8. National Institute of Justice (USA)
9. National Offender Management Service (UK)
10. National Probation Service (UK)
11. Pew Centre on the States (USA)
12. RAND Corporation
13. Swedish National Council on Crime Prevention
14. Urban Institute (USA)
15. Vera Institute of Justice (USA)
16. Washington State Institute of Public Policy (USA)
17. American Society of Criminology (USA)
18. Academy of Criminal Justice Sciences (USA)

#### 3.1.8 Additional outreach

Authors will contact a total of 30–60 experts in the field, primarily via e‐mail. The purpose of doing so is to locate unpublished studies of the effects of DRCs on recidivism. Additionally, the authors will consult with Phyllis Schultze, an information specialist at Rutgers University.

#### 3.1.9 Search strings and keywords

The keywords below will be used for searching databases and websites are detailed in Table 1. As far as possible, searches will include elements from 1, 2, and 3.

**Table 1:** Search terms to be used in systematic review query

Search element 1	Search element 2	Search element 3
Day	Program	Effective/effectiveness/effects
Evening	Report	Experiment
Daily	Centre/centre	Evaluate/evaluation
	Custody	Recidivism/recidivate
	Resource	Convict/conviction/reconviction
	Prerelease/prerelease	Arrest/rearrest/rearrest
		Incarcerate/incarceration

Since different electronic databases accept different approaches, we will create combinations of terms and, where possible, using Boolean operators (e.g., AND and OR), and wildcards (*). This will require some judgement. However, where possible, these terms will be combined in a comprehensive search strategy as follows:

(day OR evening OR daily) AND (report* OR program* OR cent* OR custod* OR resource* OR pre‐releas* OR prereleas*) AND (effective* OR experiment* OR evaluat* OR recid* OR arrest* OR incarc* OR convict*)

We will keep a record of each search, including for the keywords used and their combination, the date the search is performed, the sources consulted. For searches (e.g., through Google) that produce very large numbers of results (e.g., hundreds of thousands), attention will be given to the most relevant results that are delivered higher in the query (results will be read until no further relevant studies are identified).

### 3.2 Description of methods used in primary research

Methods used to evaluate DRCs are diverse, and we review three examples here to illustrate this diversity. We are aware of one randomized experiment focused on DRCs. This focused on seven DRCs in urban areas of New Jersey's urban areas for which parolees technically violating supervision conditions were assigned to either a DRC (*n* = 198) or intensive supervision parole condition (*n* = 204). The initial study period lasted 90 days, with data being collected for an additional 18 months afterwards. Outcome measures included completion, rearrests for new offences only, re‐convictions for new offences only, positive drug tests, employment, and time to first rearrest for a new offence.

Other studies we are aware of involve quasi‐experimental methods with varying degrees of sophistication. Solomon (2008) compared arrests of offenders sentenced to a “day custody program” (DCP; total *n* = 626) in Manhattan, New York to recidivism among a prior year's equivalent cohort (*n* = 2,231). To be eligible for DCP offenders must have had at least three prior misdemeanours, were not waiting arraignment for certain cases, have had no history of violent crime, and did not have an active hold for a warrant.

Meanwhile, Ostermann (2009) built a quasi‐experimental study comparing recidivism of New Jersey offenders paroled to halfway back programs (*n* = 181), day reporting programs (*n* = 135), parole with no programs (*n* = 198), and offenders who have maxed‐out of prison with no supervision (*n* = 200; the latter is considered the comparison group). Recidivism is measured by rearrest, reconviction, and reincarceration, and results are presented without statistical controls and with statistical controls in the form of logistic regression.

Efforts will be made to analyse randomized control trials (RCTs) and quasi‐experiments separately to account for the increased risk of selection bias in the latter type studies. However, if only one RCT is available, then studies will be compared according to whether they have RCTs or high quality quasi‐experiments on the one hand (involving quasi‐experiments with extensive design or statistical controls or recent cohort comparisons), or whether they are some other kind of quasi‐experiments on the other.

### 3.3 Criteria for determination of independent findings

Arrests, reconvictions, and reincarceration outcomes will, in the first instance, be analysed separately. Importantly, these will not necessarily be affected in the same way by DRCs, and may be a product of quite different mechanisms. This is particularly relevant to reincarceration, because this often arises in the absence of criminal behaviour, but following violation of the terms of supervision. We will continue to separate arrests reconvictions and reincarcerations given the variability in decision‐making surrounding these events. If multiple studies report effect size data for different outcomes, we will code effect sizes and conduct separate meta‐analyses for each of the different outcomes. Similarly, if multiple studies report multiple follow‐up periods, we will conduct multiple meta‐analyses, stratified by outcome (e.g., arrest, reconviction, and reincarceration) and length of follow up (e.g., 1 month, 3 months, and 6 months). We will also conduct an analysis of all studies, prioritising the most frequently occurring failure measure to maximise statistical power.

Where multiple measures are present in a single study we will choose one only, prioritising arrests because they are likely to be less dependent on downstream court‐decision‐making processes than convictions, and hence a better reflection of underlying criminal behaviour.

Where multiple studies refer to the same underlying evaluation, this will be treated as a single experiment. In such cases, there may be multiple follow‐up periods to choose between. For a given evaluation, we will choose the longest follow‐up period for which the original cohort is still largely uncompromised by attrition.

### 3.4 Details of study coding categories

Coding will be completed by the second and third authors, with the first author overseeing the coding. A full coding scheme can be found in the Appendix.

### 3.5 Statistical procedures and conventions

If there are at least two eligible studies, we will conduct meta‐analysis. We will include effect sizes based on comparable outcomes in discrete studies (e.g., rearrests and reincarceration) and calculate appropriate weights. Given the dichotomous character of the recidivism events we measure we expect our effect size measures will be log odds ratios (though we will be open to other metrics should they arise).

In experimental studies that do not include statistical controls, and where raw outcomes are reported, we will include log odds ratios based on the counts or positive and negative outcomes across treatment and control groups. However, because our selection strategy of allowing quasi‐experimental studies with statistical controls for inequalities in treatment and comparison groups, we will also likely need to include adjusted log odds ratios based on coefficients and standard errors from logistic regression analyses. If other forms of outcome measure are available, we will create appropriate effect size measures (see Lipsey & Wilson, 2001).

We will conduct random effects meta‐analysis to calculate the overall effect size, based on the assumption that variation in the observed treatment effect in part reflects real differences in the treatment between daily reporting programs (rather than sampling variability alone). We will present forest plots to visually display the effect sizes in the meta‐analysis.

We will also explore heterogeneity, for example by calculating *I* ^2^, and *Q* statistics. If appropriate (and where there are ideally at least five studies per comparison group), we will conduct a moderator analysis, to take account of program variations. Moderator analysis will use analogue to the analysis of variance (ANOVA; with random effects) to assess associations across individual categorical moderator variables and effect size variance. Moderators of interest include the population served (age, risk levels, and offence types), programing provided (intensity and type of services) and reporting regime (e.g., how frequent contacts tend to be), research design (to distinguish between stronger and weaker experimental designs), and researcher and evaluator independence (i.e., whether or not the researcher is associated with, or independent from, the day reporting centre program). Regarding the design type, if there are insufficient RCTs to make a separate comparison, we will classify studies according to whether they are RCTs or high‐quality quasi‐experiments on the one hand (involving quasi‐experiments with extensive design or statistical controls or recent cohort comparisons), or other quasi‐experiments on the other.

We will also create funnel plots to assess for publication bias, and use trim and fill methods (Duval & Tweedie, 2000) to produce adjusted mean effect size estimates (Steichen, 2000) as a robustness check to the main analysis. We plan to use user‐written programs and macros available for Stata to conduct all meta‐analysis functions (e.g., metan, metatrim, metaf.ado).

### 3.6 Treatment of qualitative research

Qualitative research studies are not included in the core review. We will, however, include relevant qualitative research in the background of our systematic review.

## ROLES AND RESPONSIBILITIES

- Content: Joel Miller, Nicole Sachs, Marina Saad
- Systematic review methods: Joel Miller, Nicole Sachs
- Statistical analysis: Joel Miller
- Information retrieval: Graduate Research Assistant (supervised by Nicole Sachs)
- Coding: Nicole Sachs, Marina Saad

## DECLARATIONS OF INTERESTS

None.

## SOURCES OF SUPPORT

Graduate Research Assistant support will be provided by a graduate student at Fairleigh Dickinson University. The student will receive credit towards her Master's degree completion for her assistance in this review.

## PRELIMINARY TIMEFRAME

Approximate date for submission of the systematic review: June 2020.

## PLANS FOR UPDATING THE REVIEW

All authors will be responsible for updating the review. Updatescan be expected every 3 years following the completion of the initialsystematic review.

## Systematic review coding protocol

**A. STUDY LEVEL CODING SHEET**

*NOTE: One study level coding sheet should be used per study. The primary publication of a study should be listed, and additional documents that list the study should be noted*.

A1: Study Identifier: studyid

A2: Cross‐reference document ID: xref1

A3: Cross‐reference document ID: xref2

A4: Cross‐reference document ID: xref3

A5: Cross‐reference document ID: xref4

A6: Coder initials: coder

A7: Date of coding: codedate

A8: Title: title

A9: Author: author

A10: Type of publication: pubtype

1 = book
2 = book chapter
3 = peer‐reviewed journal article
4 = government report (federal)
5 = government report (state/local)
6 = unpublished/gray literature
7 = other

A11: Journal reference: jref

A12: Year of publication: pubyr

A13: Date range of research: yrange

A14: Country of publication: publoc

A15: Country of study: resloc

A16: Number of treatment‐comparison contrasts in report: mods

A17: Is the same comparison group used in each contrast? compmod

0 = No
1 = Yes
99 = N/A

**B. ELIGIBILITY CHECKLIST**

B1. First author's last name: elname

B2. Coder initials: elcoder

B3. Date eligibility determined: eldate

*To be eligible, a study must meet the following criteria*.

*For B4 to B10, answer each question with 1 = Yes, 0 = No*.

B4. The study evaluates a program conducted for offenders supervised in the community. eldrc

B5. As part of the program, the offender is required to make appearances at a physical premises on multiple (more than one) occasions each week. elsup

B6. The study includes a comparison group receiving ‘standard probation/parole,’ not comprised of dropouts from DRC, or other supervision by community corrections official (not incarcerated controls), or incarceration disposition. Study design may be experimental or quasi‐experimental, but not a one‐group research design. elcomp

B7. The study includes a post‐program measure of criminal behavior (new arrests, convictions, and/or incarcerations). May be official or self‐reported and dichotomous or continuous. elrecid

*For documents that do not meet the above criteria, answer the following questions*:

B8. Document is not a quantitative evaluation (no data regarding effects of DRC reported). noquant

B9. Document is a review article relevant to this project (e.g., references to studies, background information for write‐up). elrev

B10. Document status: elstat

0 = Not eligible
1 = Eligible
99 = Relevant review article

**C. TREATMENT‐COMPARISON CODING SHEET**

*NOTE*: *If the study reports on multiple treatment‐comparison contrasts, or multiple treatments compared to a single comparison group, each contrast should be coded on separate Treatment‐Comparison Coding Sheets. Only independent evaluations should be included in analyses (i.e., multiple treatment groups should not have overlapping participants)*.

Identifying information

C1. Study identifier: studid

C2. Module ID: modid

C3. Coder initials: txmod

Program details

C4. Description of what happens to treatment group: txdesc

C5. Description of what happens to control group: cxldesc

C6. What was the precise nature of the program? progdesc

1 = ‘Front door’ prison diversion (DRC instead of prison)
2 = ‘Backdoor’ prison diversion (early release from prison)
3 = Other

C6a. Primary program components (indicate whether present or not): progcomp

Number of days per week to report to DRC: drcday
Number of hours per day to report to DRC: hourday
Number of hours per week to report to DRC: hourwk
Program increases frequency of contact with probation/parole officer: progdf
  0 = No
  1 = Yes
Program increases drug testing requirements: progdt
  0 = No
  1 = Yes
Program includes substance abuse counseling services: dtxserv
  0 = No
  1 = Yes
Program includes mental health services: mhserv
  0 = No
  1 = Yes
Program includes employment services: empserv
  0 = No
  1 = Yes
Program includes life skills training: skillserv
  0 = No
  1 = Yes
Program includes other services: othserv
  0 = No
  1 = Yes

C6b. If Yes for any of the above, state exact numbers if available (999 if not):

Control ratio: / Treatment ratio: racxl/ratx
Control freq: / Treatment freq: frcxl/frtx
Control drug tests: / Treatment drug tests: drcxl/drtx

C6d. Additional program components (0 = No, 1 = Yes):

Curfew: curf
Electronic monitoring: elecm
Offense‐specific treatment: offtx (e.g., sex offender treatment)

C7. What happened to the comparison group? cxltype

1 = “Supervision as usual”
2 = “Incarceration”
99 = Other

C8. Was supervision for treatment group provided by anyone other than probation/parole officer? posup

0 = No
1 = Yes
99 = Don't know/can't tell

C9. Length of DRC intervention in months

Minimum: txlmin
Maximum: txlmax
Mean: txlmn
Fixed (same for all subjects):txlfix

C10. Did the intervention follow a set protocol? txprot

0 = No
1 = Yes
99 = Don't know/can't tell

C11. What supervision philosophy was stated? txphil

1 = Control/surveillance
2 = Treatment
3 = Hybrid
4 = Other
99 = Don't know

C12. Did the intervention remain consistent over time? txcons

0 = No
1 = Yes
99 = Don't know/can't tell

Methodological Rigor

C13. Control variables used in statistical analyses to account for initial group differences? cxlvars

0 = No
1 = Yes

C14. Subject‐level matching? Matching

0 = No
1 = Yes

C15. Random assignment to conditions? Random

0 = No
1 = Yes

C16. Measurement of prior criminal involvement? prior

0 = No
1 = Yes

C17. Rating of initial similarity between treatment and control group: prsim

**Table:**

1	2	3	4	5	6	7

(1 = Nonrandomized; high likelihood of baseline differences between groups or known differences related to future recidivism) (5 = Nonrandomized design with strong evidence of initial equivalence) (7 = Randomized design with large N or small N design with matching)

C18. Was attrition discussed in the report? attrep

0 = No
1 = Yes

C19. Is there a potential threat to generalizability from overall attrition? attgen

0 = No
1 = Yes

C20. Is there a potential threat to internal validity from differential attrition? attint

0 = No
1 = Yes

C21. Did the statistical analysis attempt to control for differential attrition effects? attstat

0 = No
1 = Yes
99 = Don't know/can't tell

C22. Statistical significance testing used? sigtest

0 = No
1 = Yes

C23. Overall methodology rating: methrat

1 = Comparison group(s) lack demonstrated/plausible comparability to treatment group, no controls (nonrandom assignment)
2 = Comparison group(s) lack demonstrated/plausible comparability to treatment group, credible statistical controls used (nonrandom assignment)
3 = Comparison groups demonstrably/plausibly similar between groups, no statistical controls (nonrandom assignment)
4 = Comparison groups demonstrably/plausibly similar between groups, credible statistical controls used (nonrandom assignment)
5 = Random assignment and analysis of comparable program and comparison groups, notably attrition (not controlled)
6 = Random assignment and analysis of comparable program and comparison groups, notably attrition (controlled)
7 = Random assignment and analysis of comparable program and comparison groups, no/limited attrition

Notes on methodology:

**D. SAMPLE LEVEL CODING SHEET**

*NOTE: A study may report results separately for distinct samples (e.g., persons with/without prior arrests). Each distinct sample must have its own coding sheet. The treatment‐comparison contrast is the same for the different samples. Samples should be independent; i.e., no overlapping participants. Some studies report the results broken down by different subgroups (e.g., by gender). Only one of these breakouts can be used—choose the one with the most information, or the one most relevant to the review*.

Identifying information

D1. Study identifier: studid

D2. Module ID: modid

D3. Sample ID: sampid D4.

D4. Coder initials: codersamp

Sample description

D5. Description of treatment group sample: txsamp

D6. Description of comparison group sample: cxlsamp

D7. Total N in treatment group at beginning of study: txn

D8. Total N in comparison group at beginning of study: cxln

*Note: D7* + D8 = total sample size before attrition. If multiple samples are being coded, the sum across samples must equal the total sample size before attrition.

D9. Age range of study participants: sampage

1 = Adolescent (12‐18)
2 = Adult (18+)
3 = Adolescent and adult
4 = Other
99 = Unspecified/can't tell

D10. Youngest age included in sample (999 if unknown): yage

D11. Oldest age included in sample (999 if unknown): oage

D12. Exact proportion of males in sample (if known): prmale

D13. Approximate gender description of sample: sampgen

1 = All male (>90%)
2 = More males than females
3 = All female (>90%)
4 = More females than males
5 = Roughly equal males and females
99 = Can't tell

D14. Race/ethnicity of sample (999 if unknown):

% Asian: rasian
% Native American: rnative
% Black: rblack
% White: rwhite
% Hispanic: rhisp
% Mixed: rmixed
% Other: rother

D15. General offender type: offtype

1 = Violent and/or person crimes
2 = Nonviolent and/or nonperson crimes
3 = Mixed: violent/nonviolent
4 = Specialized caseload: drugs
5 = Specialized caseload: sex offenders
6 = Specialized caseload: mental
7 = Specialized caseload: domestic violence
8 = Other
9 = Don't know/can't tell

D16. Composition of supervised offenders: offcomp

1 = All probationers
2 = All parolees
3 = Probationers and parolees

D16a. If combination of probationers and parolees (999 if unknown):

% probation: pcpro
% parole: pcpar

D17. Probationer/parolee risk level: offrisk

1 = Low risk
2 = Medium risk
3 = High risk
4 = Low and medium risks
5 = Medium and high risks
6 = All risk levels
7 = Other
8 = Don't know/can't tell

D18. How was risk determined? riskjmt

1 = Statistical model
2 = Prior convictions
3 = Instant offense
4 = Judgment of probation/parole officer
5 = Classification instrument
6 = Other
7 = N/A
99 = Don't know/can't tell

D19. Probationer/parolee need level: offneed

1 = Low need
2 = Medium need
3 = High need
4 = Low and medium need
5 = Medium and high need
6 = All need levels
7 = No need assessment
8 = Other
99 = Don't know/can't tell

**E. DEPENDENT VARIABLE CODING SHEET**

*NOTE*: *Code each dependent variable reported in the study separately. The same dependent variable measured at multiple times should be coded only once. For non‐crime outcomes, code only items E6, E9, and E10*.

Identifying information

E1. Study identifier: studid

E2. Module ID: modid

E3. Sample ID: sampid

E4. Outcome ID: outid

E5. Coder initials: coout

Outcome information

E6. Outcome label (label used in the report): outlab

E7. Recidivism construct represented by this measure: rconst

1 = Arrest
2 = Charge
3 = Conviction
4 = Technical violation
5 = Probation/parole
6 = Incarceration
7 = Other

E8. Offense types included in recidivism measure:

All offenses (“No” for others): offenses
  0 = No
  1 = Yes
Drug offenses: odrug
  0 = No
  1 = Yes
Person offenses, sexual: opsx
  0 = No
  1 = Yes
Person offenses, nonsexual: opnsx
  0 = No
  1 = Yes
Person offenses, unspecified: opuns
  0 = No
  1 = Yes
Property offenses: oprop
  0 = No
  1 = Yes
Weapons offenses: oweap
  0 = No
  1 = Yes
Driving offenses: odrive
  0 = No
  1 = Yes
Technical or status offenses: otech
  0 = No
  1 = Yes
Other: oother
  0 = No
  1 = Yes

E9. Measurement scale: mscale

1 = Dichotomous
2 = Trichotomous
3 = 4‐9 discrete ordinal categories
4 = >9 discrete ordinal categories/continuous

E10. Source of data: dsrce

1 = Self‐report
2 = Other report (e.g. parole officer)
3 = Official records
4 = Other
99 = Don't know/can't tell

E11. Length of follow‐up period: fulng

1 = < 6 months
2 = 6‐12 months
3 = >1, <2 years
4 = > 2 years
5 = No follow‐up
99 = Don't know/can't tell

E12. Is cost/benefit data for the program included in the study?

0 = No
1 = Yes

**F. EFFECT SIZE LEVEL CODING SHEET**

*NOTE*: *Complete a separate coding sheet for each treatment‐comparison contrast for each dependent variable*.

Identifying information

F1. Study identifier: studid

F2. Module ID: modid

F3. Sample ID: sampid

F4. Outcome ID: outid

F5. Effect size ID: esid

F6. Coder initials: coes

Effect size information

F7. Effect size type: estype

1 = Baseline (pretest; before start of intervention)
2 = Post‐test (first measurement point, postintervention)
3 = Follow‐up (all subsequent measurement points, postintervention)

F8. Which group does the raw effect favor (ignoring statistical significance)? esdir

1 = Treatment group
2 = Comparison group
3 = Neither (ES = 0)
99 = Can't tell (ES cannot be used if selected)

F9. Does the investigator report the difference as statistically significant? essig

0 = No
1 = Yes
2. Not tested
99. Can't tell

F10. If tested, what type of statistical test was used? estest

1 = *t* test
2 = *F* test
3 = χ^2^
4 = Regression analysis
5 = Other
6 = N/A
99 = Can't tell

F11. Timeframe in months captured by the measure

Minimum: estmin
Fixed (same for all subjects): estfix
Maximum: estmax
Mean:estmn

F12. Timeframe in months from end of program to measurement point

Minimum: esfumin Fixed (same for all subjects): esfufix Maximum: esfumax Mean:esfumn

Effect size data—all effects

F13. Treatment group sample size for this ES: estxn

F14. Comparison group sample size for this ES: escxl

Effect size data—continuous outcomes

F15. Treatment group mean: estxmn

F16. Comparison group mean: escxlmn

F17. Are the above means adjusted? esmadj

0 = No
1 = Yes

F18. Treatment group standard deviation: estxsd

F19. Comparison group standard deviation: escxlsd

F20. Treatment group standard error: estxse

F21. Comparison group standard error: escxlse

F22. *t* value from an independent t‐test or square root of *F* value from a one‐way ANOVA with 1 *df* in the numerator (only 2 groups): estval

F23. Exact probability for a *t* value from an independent *t* test or *F* value from a one‐way ANOVA with 1 *df* in the numerator: estvalp

F24. Correlation coefficient: escorr

Effect size data—dichotomous outcomes

F25. Number successful in treatment group: estxs

F26. Number successful in comparison group: escxls

F27. Proportion successful in treatment group: estxspr

F28. Proportion successful in comparison group: escxlspr

F29. Are the above proportions adjusted for pretest variables? espradj

0 = No
1 = Yes

F30. Logged odds ratio: eslogor

F31. Standard error of logged odds ratio: eslorse

F32. Logged odds ratio adjusted? (e.g., from logistic regression) esloradj

0 = No
1 = Yes

F33. χ^2^ value with 1 *df* (2 × 2 contingency table): eschisq

F34. Correlation coefficient: esdcorr

Effect size data—hand calculated

F35. Hand calculated d‐type effect size: eshand

F36. Hand calculated SE of the d‐type effect size:eshandse

*Coding protocol adopted from Gill, Hyatt, and Sherman (2010).
